# CXCL12-CXCR4/CXCR7 Axis in Colorectal Cancer: Therapeutic Target in Preclinical and Clinical Studies

**DOI:** 10.3390/ijms22147371

**Published:** 2021-07-09

**Authors:** Tripti Khare, Marc Bissonnette, Sharad Khare

**Affiliations:** 1 Division of Gastroenterology and Hepatology, Department of Medicine, University of Missouri, Columbia, MO 65212, USA; kharet@health.missouri.edu; 2 Section of Gastroenterology, Hepatology and Nutrition, Department of Medicine, University of Chicago, Chicago, IL 60637, USA; mbisssonn@medicine.bsd.uchicago.edu; 3 Harry S. Truman Memorial Veterans’ Hospital, Columbia, MO 65201, USA

**Keywords:** colorectal cancer, metastasis, CXCL12, CXCR4, CXCR7, therapeutics

## Abstract

Chemokines are chemotactic cytokines that promote cancer growth, metastasis, and regulate resistance to chemotherapy. Stromal cell-derived factor 1 (SDF1) also known as C-X-C motif chemokine 12 (CXCL12), a prognostic factor, is an extracellular homeostatic chemokine that is the natural ligand for chemokine receptors C-X-C chemokine receptor type 4 (CXCR4), also known as fusin or cluster of differentiation 184 (CD184) and chemokine receptor type 7 (CXCR7). CXCR4 is the most widely expressed rhodopsin-like G protein coupled chemokine receptor (GPCR). The CXCL12–CXCR4 axis is involved in tumor growth, invasion, angiogenesis, and metastasis in colorectal cancer (CRC). CXCR7, recently termed as atypical chemokine receptor 3 (ACKR3), is amongst the G protein coupled cell surface receptor family that is also commonly expressed in a large variety of cancer cells. CXCR7, like CXCR4, regulates immunity, angiogenesis, stem cell trafficking, cell growth and organ-specific metastases. CXCR4 and CXCR7 are expressed individually or together, depending on the tumor type. When expressed together, CXCR4 and CXCR7 can form homo- or hetero-dimers. Homo- and hetero-dimerization of CXCL12 and its receptors CXCR4 and CXCR7 alter their signaling activity. Only few drugs have been approved for clinical use targeting CXCL12-CXCR4/CXCR7 axis. Several CXCR4 inhibitors are in clinical trials for solid tumor treatment with limited success whereas CXCR7-specific inhibitors are still in preclinical studies for CRC. This review focuses on current knowledge of chemokine CXCL12 and its receptors CXCR4 and CXCR7, with emphasis on targeting the CXCL12–CXCR4/CXCR7 axis as a treatment strategy for CRC.

## 1. Introduction

The incidence and mortality rates of colorectal cancer (CRC) vary remarkably around the world. CRC is the third most common cancer diagnosed and fourth most common cause of death in males, in contrast, this malignancy being the second most diagnosed and third most common cause of death in females [1,2]. Despite decreasing mortality globally, there is increased incidence and mortality rate amongst patients younger than 50 years of age. Usually when diagnosed initially, almost 25% of patients have metastatic CRC (mCRC) and half of the individuals undergoing tumor resection later develop metastasis with a 5-year survival rate ranging from 27% to 58% [3,4]. Thus, despite significant advances in managing CRC, the 5-year survival rate for patients with mCRC is disappointingly low. The pathogenesis of CRC is complex as it is influenced by lifestyle-related factors, for example, smoking and alcohol use, poor diet, sedentary lifestyle, obesity, environmental factors, as well as genetic predisposition, for example, families with increased colon polyps or an inflammatory bowel such as Crohn’s disease and ulcerative colitis. Although most colon cancers are sporadic, inherited germline mutations account for less than 10% of CRC [5].

Cancers are comprised of transformed cells and non-malignant cells including cancer-associated fibroblasts (CAF), endothelial vasculature cells and tumor infiltrating cells (T-cells, macrophages, and neutrophils). These nonmalignant cells, as well as soluble factors (cytokines and growth factors (GF)), and the extracellular matrix form the tumor microenvironment (TME) [6,7]. In general, the cancer cells and their surrounding TME crosstalk by direct cell to cell contact and via soluble factors, such as cytokines (GFs and chemokines). TME not only promotes cancer progression through growth promoting cytokines, but also provides resistance to chemotherapy [8]. New cancer therapies have targeted TME and tumor–stroma interactions (TSI). Understanding the mechanisms of tumor progression and roles of cytokines in CRC will likely suggest new therapeutic targets. Recent review articles related to C-X-C motif chemokine ligand 12 (CXCL12)-chemokine receptor type 4 (CXCR4)/ chemokine receptor type 7 (CXCR7) axis are focused on cancers in general, however, our focus in this review is to summarize the role of this axis in CRC progression and metastasis. Significant inhibitors or antagonists of this axis in preclinical and clinical studies of gastrointestinal cancers are also discussed. 

We performed a literature search in PubMed and Google Scholar including the terms “Cytokines”, “Chemokines”, “CXCL12“, “Colorectal Cancer”, and “Metastasis” that resulted in 82 and 250 hits, respectively, in the last 10 years. References were selected with emphasis on CXCL12, CXCR4, CXCR7 in CRC. References from other solid tumors and hematologic malignancies are mostly not included. 

## 2. Cytokines, Chemokines, and Receptors

Cytokines are soluble proteins (~5–20 kDa), which are released from cancer cells, as well as stromal cells in response to environmental cues. Cytokines, on binding to their cognate membrane receptors, activate several signal transduction pathways regulating complex physiological and pathological processes. Cytokines include families of growth factors, chemokines, angiogenic factors, and interferons that originate from cancer cells, immune cells such as macrophages, dendritic cells, T and B lymphocytes, and sentinel cells that cross talk with immune cells, such as fibroblasts, and endothelial cells [9]. 

Chemokines are an important class of cytokines with major roles in regulating the TME and resistance to cancer treatment. Chemokines were initially characterized through their ability to cause the migration of leukocytes. They are 8–10 kDa secreted polypeptides that regulate inflammatory processes in tissue environments [10]. Many chemokines are promiscuous and can bind to more than one receptor and chemokine receptors, in turn, can frequently bind to multiple chemokines [11,12]. Paracrine and autocrine signaling by chemokines generate complex communications between tumor cells and TME [13]. Different types of chemokines and seven-transmembrane-spanning G protein-coupled receptors (GPCR) have been demonstrated in humans and reported to be responsible for several aspects of intercellular interactions [14,15]. The chemokines are divided into four families (CC, CXC, CX3C, and XC) based on the arrangement of the two cysteine residues near the amino terminus [16]. 

Chemokine receptors have their N-terminus outside the cell and the C-terminus in the cytoplasm. In addition, there are three extracellular and three intracellular loops with seven transmembrane helices [17]. Coupling of one of the intracellular loops of the receptor to G-proteins mediates receptor binding to the ligand followed by activation of a series of signal transduction pathways [18]. Based on their chemokine ligands, chemokine receptors are divided into two subfamilies: conventional chemokine receptors (cCKR) and atypical chemokine receptors (ACKR) [19]. Chemokine binding to a cCKR and ACKRs induces a conformational change in the receptor, leading to activation of MAPK/ERK, PI3K/AKT, and JAK/STAT signaling pathways [20,21,22,23,24,25,26]. Recently, in addition to canonical chemokines receptors, four ACKRs have been identified [19]. Structures of ACKRs are highly homologous to conventional GPCRs, except that the atypical receptors do not induce chemotaxis [19]. Rather, these ACKRs regulate intracellular storage, extracellular bioavailability, and distribution of chemokines in polarized cells [27].

## 3. Chemokine CXCL12

CXC chemokine CXCL12, also known as stromal cell-derived factor-1 (sdf-1), is an extracellular homeostatic chemokine that binds to CXCR4 (CD184), and CXCR7 [28,29]. A major function of CXCL12 is to regulate trafficking of hematopoietic cells and architecture of secondary lymphoid tissues. The chemotactic cytokine CXCL12 is expressed constitutively in lung, bone marrow, and liver, the most common sites for colon cancer metastasis [12]. CXCL12 is also expressed constitutively in several other organs, such as skeletal muscle, brain, kidney, heart, and skin [12]. The migration of cancer cells to these organs is dependent on CXCL12 expression [30]. The gene coding for human CXCL12 is located on chromosome 10q11 [31]. It is the only CXC chemokine with differential mRNA splicing. Single CXCL12 gene encodes six protein isoforms in human (CXCL12α to ϕ) and three isoforms in mouse (CXCL12α to γ) by alternate splicing of the fourth and final exon. These isoforms are differentially expressed and have differential affinity to cell surface and extracellular matrix glycosaminoglycans [32]. The meta-analysis of CXCL12 expression revealed that it can be used as prognostic biomarker for various cancer types [33]. Akishima-fukasawa et al. investigated the expression level of CXCL12 in CRC patients and demonstrated that CXCL12 expression in CRCs and at sites of budding is a promising prognostic factor [34]. 

CXC chemokines are sub-classified as promoters or suppressors of angiogenesis based on the presence or absence of ELR(Glu-Leu-Arg) motif, respectively. Specifically, the ELR-positive chemokines (CXCL1, CXCL6, and CXCL8) promote angiogenesis, whereas ELR-negative chemokines (CXCL4, CXCL10, and CXCL14) inhibit angiogenesis [35]. CXCL12 (sdf-1) is also an ELR-negative chemokine. However, unlike other ELR- negative chemokines, CXCL12 is one of the most potent angiogenesis-promoting chemokines [36]. Mice with defective CXCR4 exhibit impaired vascular formation, which, according to this classification, does not fit with activities assigned to ELR-negative chemokines [37]. Moreover, CXC chemokines can be further classified as inflammatory, homeostatic, or dual function according to their role in immune system and inflammatory response [38]. CXCL12 belongs to the homeostatic group.

A key role of chemokine CXCL12 and its receptors is in regulating metastasis by multiple mechanisms including facilitating tumor spread, affecting endothelial adhesion, eruption from blood vessels, proliferation, angiogenesis, colonization, and evasion from host response via activation of key survival pathways regulated by ERK/MAPK, Jak/STAT, and PI3K/Akt/mTOR signaling [39]. In addition, CXCL12 regulates the expression and function of integrins required for cell adhesion [40]. Additionally, chemokines promote communication between cancer cells and the surrounding non-neoplastic cells in the TME, which include endothelial cells and fibroblasts. Chemokines also increase infiltration, and activation of neutrophils and tumor associated macrophages (TAM) [41]. 

## 4. Chemokine Receptors

### 4.1. CXCR4

CXC chemokine receptor 4 (CXCR4), a 352 amino acid rhodopsin-like GPCR was first investigated as chemokine receptor associated with breast cancer metastasis to the lung tissues [17]. CXCR4 is the most widely expressed chemokine receptor among more than 23 human cancers, including breast, ovary, melanoma, prostate, and CRC, though CXCR4 expression is low or absent in many normal tissues [42,43,44]. In CRC, the expression of CXCR4 in primary tumor cells is associated with recurrence, metastasis, and survival [45]. Mechanisms of CXCR4 gene upregulation in CRC are not well understood. Aberrant cancer signaling may result in CXCR4 overexpression. We reported, for the first time, that 5-methylcytosine (5mC) distribution in the CXCR4 promoter is not significantly altered in primary colon cancers. However, increased CXCR4 expression in CRC is associated with enhanced 5-hydroxymethylcytosine (5hmC) deposition in the gene body [46]. 

CXCR4 can exist as a monomer, dimer, oligomer, or nanoclusters in the plasma membrane [47]. CXCL12 is canonical ligand of CXCR4. Macrophage migration inhibitory factor (MIF) and extracellular ubiquitin, however, can also activate CXCR4 [48,49]. CXCR4 is not only involved in cancer metastasis but can also modulate other functions of cancer stem cells [50]. CXCL12–CXCR4 is the best characterized axis involved in homing and retention of hematopoietic stem cells (HSC) in the bone marrow [51]. In this regard, the Food and Drug Administration (FDA) has approved the drug plerixafor, a competitive inhibitor of CXCR4 for HSC transplantation. Blocking the interaction of CXCL12–CXCR4 results in mobilization of CD34 and HSCs to the peripheral blood [52,53]. Moreover, involvement of CXCL12–CXCR4 axis in tumor growth, invasion, angiogenesis, and metastasis in a variety of cancer types, such as breast, CRC, and pancreatic cancer has been documented by several researchers [54,55,56]. Expression of CXCR4 correlates well with recurrence, metastasis, and survival in melanoma and CRC [45]. However, CXCL12–CXCR4 axis has less of a role to play in locating melanoma and colorectal tumors to the bone marrow as bone metastasis is not as high as reported in other cancers like prostate and breast [57]. Mechanistically, binding of CXCR4 to chemokine CXCL12 leads to activation of G proteins. After activation, G proteins dissociate into Gα regulating RAS/RAF and Gβγ activating P13K/Akt/mTOR and MEK/ERK. Additionally, CXCL12–CXCR4 axis also regulates apoptosis via involvement of Bcl-2 family members [58]. 

### 4.2. CXCR7

Chemokine CXC receptor 7 (CXCR7), recently termed ACKR3, is amongst the G-protein-coupled cell surface receptor family that is commonly expressed in a large variety of cancer cells. Various inconsistencies in human cancer cell lines with varied levels of CXCR4 expression as well as studies with CXCR4 knock out mice challenged the paradigm of CXCL12 binding to CXCR4 [59]. These findings suggested the existence of another CXCL12 receptor that was identified in 2005 as CXCR7 [60]. It was first cloned from thyroid cDNA library of dog and, therefore, named as receptor dog cDNA-1(RDC-1) [61]. The genes coding for CXCR1, CXCR2, CXCR4, and CXCR7 are all located on mouse chromosome 1 and human chromosome 2. CXCR7 is suggested to be a marker of memory B cells, destined to become antibody secreting cells as suggested by its correlated expression with B cells that can differentiate into plasma cells upon activation [62].

CXCR7 is reported to be highly expressed in many tumors and tumor-associated blood vessels including cancers of liver, colon, pancreas, prostate, and lungs [63,64,65]. CXCL12 binds CXCR7 with higher affinity than it binds CXCR4. Recent studies have suggested that CXCR7, like CXCR4, is important for cancer cell survival, migration, adhesion, angiogenesis, and metastasis [59,66]. However, unlike CXCR4, CXCR7, a non-classical GPCR, cannot stimulate G proteins. CXCR7 possesses a slight modification at two positions (A/S and V/T) in the canonical DRYLAIV motif and does not increase intracellular Ca^2+^ release after binding to CXCL12, an essential step in the recruitment and activation of G proteins [67]. Due to this discrepancy many mechanisms underlying CXCR7 functions have been suggested. For example, CXCR7 as a scavenger receptor is responsible for creating a gradient of CXCL12 and, thereby, influencing cell migration through CXCL12-CXCR7 internalization [60,68]. Another hypothesis is that CXCR7 functions at least in part by recruiting β-arrestin2, heterodimerizing with CXCR4, and acting as a “scavenger” of CXCL12, thereby lowering the level of CXCL12 and weakening the activity of CXCR4 [69]. There is also evidence that non-G-protein mediated accumulation of β-arrestin2 induced by CXCL12-CXCR7 binding activates extracellular regulated protein kinase (ERK) through mitogen activated protein kinase (MAPK) signaling [70]. CXCR7 can also bind with almost 10-fold lower affinity to CXCL11 (interferon-inducible T cell alpha chemoattractant(I-TAC)), another chemokine, to recruit CXCR4 [59,71]. CXCL11/I-TAC binds with high affinity to its receptor CXCR3, which is found mainly in leukocytes and expressed in many tissues including brain, pancreas, lung, and spleen [72]. Although binding affinity of CXCL11 to CXCR7 is low, but it can induce intracellular signaling in CXCR3-negative tumor and non-tumor cells [73].

## 5. Signaling in CRC Growth, Invasion, and Metastasis

### 5.1. CXCL12–CXCR4 Axis

Surgical resection of localized CRC is curative, but patient survival is diminished if the tumor has metastasized to distant organs such as liver or lungs. The 5-year survival rate with localized CRC is approximately 80–90% whereas it is only 27–58% in patients with distant metastasis [3,74]. Metastasis, the key cause of death among cancer patients, usually involves multiple genetic and epigenetic changes in the tumor. Increased expression and the tumor promoting role of the CXCL12–CXCR4 axis has been already established in several tumors, both in vitro and in vivo. These include kidney, lung, brain, prostate, colon, breast, pancreas, ovarian tumors, and endometrial cancer [43,56,75,76,77,78,79,80]. Increased trafficking of leukocytes to tumor tissues, as well as increased expression of chemokine receptors associated with poor prognosis, were reported in several solid tumors including melanomas, kidney, lung, brain, prostate, pancreas, CRC, and ovarian tumors [43,81,82,83]. A hypoxic tumor microenvironment favors up-regulation of CXCR4 and CXCL12 in monocytes, monocyte-derived macrophages, tumor-associated macrophages, endothelial cells, and cancer cells through a global regulator hypoxia-inducible factor-1 alpha (HIF-1α) [84]. This axis is also up-regulated by specific microRNAs (miR), for example, miR-126 in CRC [85]. CXCL12-CXCR4 participates in metastasis of CRC [86], melanoma [87], pancreatic cancer [88,89], hepatocellular carcinoma [90], and breast cancer [43]. Moreover, increased hepatocyte CXCL12 expression is associated with increased CXCR4 (+) cells in melanoma and CRC liver metastasis presumably by promoting cancer cell invasion [45]. Additionally, colon cancer cell invasion and EMT involves CXCL12-CXCR4 axis via Wnt/β-catenin signaling [91]. Consistent with a tumor promoting role of CXCL12–CXCR4 axis, CXCR4 neutralizing antibodies or CXCR4 knock down with shRNA reduces tumor cell invasion [92]. In brief, the CXCL12–CXCR4 axis is involved in the process of tumorigenesis and metastasis in CRC.

### 5.2. CXCL12-CXCR7 Axis

There are inconsistent reports of CXCR7 expression levels in several CRC cell lines such as RKO, HCT116, SW480, CT26, HT29, and CaCO_2_ [93,94,95,96]. SW480 cells transduced with CXCR7 significantly upregulated genes related to lipid and fatty acid metabolism [97]. CXCR7 is usually expressed in the cytoplasm of CRC cells [98], however, HT29 cells exhibits its expression in the cytomembrane [95]. Xu H et al. reported a potential role of CXCR7 in CRC development, growth, and metastasis involving TLR4 signaling [96]. Song ZV et al. documented that villin-dependent CXCR7–CXCR4 up-regulated mice exhibited more severe colitis and tumorigenesis than villin-CXCR4 or villin-CXCR7 alone up-regulated mice when exposed to dextran sodium sulfate (DSS) [99]. In human CRC, silencing of CXCR7 gene induces cell apoptosis and inhibition of CRC through ERK1/2 and β-arrestin2 pathways regulating MMP-2 and caspase-3 expression [100]. CXCR7 regulates CRC growth independent of CXCL12, but binds to CXCL12 for tumorigenesis and metastasis [101]. CXCR7 induces migration of colon cancer HT29 and SW480 cells in vitro [95,96]. However, its silencing did not affect migration, as well as activation of PI3K/Akt or ERK/Ras signaling pathways. It may affect migration indirectly by enhancing cell adhesion [93]. In SW480 and Colo 205 colon cancer cells, LPS elevated CXCR7 and increased proliferation and migration but not CXCR4 [96]. Thus, chemokine receptors CXCR4 and CXCR7 appear to play different roles in CRC. 

There are significant associations among CXCR7 expression and lymph node metastasis [96], lung metastasis [102], and tumor node metastasis (TNM) stages in CRC [98]. The expression level of CXCR7 in lung tissues is twelve times higher than in liver tissue [93,103]. Increased expression of CXCR7 was also reported in CRC with lymph node metastasis and considered a good predictor of lymph node metastasis in CRC patients [104]. The systemic treatment with CXCR7 antagonists reduces tumor expansion in lungs of experimental mice inoculated with HT-29 or C26 cancer cells [103]. Secretion of its ligand CXCL12 in cancer cells is sufficient to cause paracrine release in lungs but not in liver, thereby promoting progression of metastasis and worsening of the disease. CRC metastasis in lungs also exhibit increased CXCR7 expression in vascular endothelium of lung tissues. Binding of CXCR7 to CXCL12 directly or indirectly regulates increased development of blood vessels in lung metastasis. CXCR7 antagonists inhibited lung but not liver metastasis of CT26 cells and HT29 cells [103]. CXCR7 also regulates CRC angiogenesis independent of CXCL12 by modulating expression of VEGF via AKT and ERK pathways [101]. Unlike CXCR4, the expression of CXCR7 is not regulated by hypoxia or HIF-1α in colon cancer cell lines [93]. 

### 5.3. CXCR4 and CXCR7 Interactions

In some tumors, only CXCR4 or CXCR7 is expressed, whereas in other tumors both cytokine receptors are expressed. When concomitantly expressed, CXCR4 and CXCR7 can form homo- or hetero-dimers. The presence of CXCR7/CXCR4 heterodimers was detected in 65% of colon cancers by confocal imaging. The cross talk between CXCR4 and CXCR7 for binding to CXCL12 is mediated through intracellular signaling effectors like β-arrestin2 [105]. Villin promoter-dependent overexpression of the transgene CXCR4 and CXCR7 increased colitis and tumorigenesis compared to over expression of CXCR4 or CXCR7 alone in mouse models [99]. Similarly, CXCL12 increased phosphorylation of p38 MAPK and SAPK in cancer cells co-expressing CXCR4 and CXCR7 [106]. Because of altered CXCR4 and Gαi spatial orientation, heterodimerization of CXCR4 and CXCR7 results in modified Gαi signaling, decreased intracellular calcium release and increased β-arrestin2 signaling [107]. Heterodimers form complex with β-arrestin2 [99] followed by prolonged ERK1/2 and p38 MAPK signaling with increased chemotaxis towards CXCL12 [106]. Moreover, CXCR4 and CXCR7 co-expression synergistically enhance the recruitment of β-arrestin2 and activation of ERK1/2 and p38 MAPK signaling to increase invasion and metastasis. The heterodimers also enhance infiltration of M-myeloid-derived suppressor cells and M2-like macrophages in colonic tissues [99]. However, binding of CXCR7 to CXCL12 results in inhibition of CXCR4-mediated phosphatidylinositol-3 phosphate (PI3K)/MAPK signaling [99,106,108]. 

Increased histone demethylation by lysine demethylase activation contributes to colorectal tumorigenesis by CXCR4/CXCR7 heterodimers. The increased level of JMJD2A induced by CXCR4/CXCR7 heterodimers results in demethylation of histones H3K9me3 and H3K36me3. Moreover, demethylation of H3K9me3 and H3K36me3 histone by monomers of CXCR4 or CXCR7 is not as strong as that induced by CXCR4/CXCR7 heterodimers [99]. Differential signals are also reported regarding CXCR4/CXCR7 signaling, indicating the need for more studies to delineate signals induced by homo and heterodimers [97]. In summary, the above findings indicate that heterodimerization of CXCR4 and CXCR7 results in altered and synergistic signaling with increased colorectal tumorigenesis and metastasis. 

## 6. Therapeutics in Preclinical and Clinical Trials

Based on recent research that the CXCL12–CXCR4/CXCR7 axis is involved in survival, tumor growth, angiogenesis, metastasis, TME, and drug resistance, it is considered a promising target for therapeutic intervention. TME and TSI contribute towards drug resistance observed in cancer cells and better understanding of mechanisms driving drug resistance is needed to advance therapy [109]. Chemokines, an important component of TME, drive tumor-specific immune responses, and promote invasion, metastasis, stemness, and resistance to chemo- and radiotherapy [110,111]. Although the CXCL12–CXCR4/CXCR7 axis is considered a potential target for cancer therapies, very little is known about its distribution and activity in TME. Matsusaka S et al. reported that single-nucleotide polymorphism in CXCR4 predicts progression-free survival in mCRC patients and may help clinicians make therapeutic decisions in mCRC patients receiving first-line bevacizumab-based chemotherapy [112]. Ottaiano A et al. reported expression of CXCR4 in the primary tumor as a prognostic factor and corelated expression levels with response to first-line chemotherapy in mCRC patients [113]. 

A recent report revealed that CXCL12 and relative expression of CXCL12–CXCR4 axis are independent prognostic factors for 5-year disease-free survival [114]. However, only few drugs have been approved for clinical use targeting this axis. Multiple antagonists of CXCR4 including bicyclams (AMD3100) and T22, as well as peptide analogues designed for the N-terminal region of CXCL12, such as TN14003, CTCE-9908, and ALX40-4C are currently used to target CXCR4 in various cancers [115,116,117]. CTCE-9908, a 17 amino acid peptide analog of CXCL12 has CXCR4 antagonistic activity and phase I/II trial involving CTCE-9908 was completed in patients with advanced solid tumors [118]. CTCE-9908 blocks binding of CXCR4 to CXCL12, a critically important event involved in infiltration of organ tissues by metastatic cells. CXCR4 antagonist AMD3100 (Plerixafor or Mozobil) is the most frequently used drug in clinical trials, which targets the CXCL12–CXCR4/CXCR7 axis [119]. Peng SB et al. reported antitumor effects of CXCR4 inhibitor LY2510924 in CRC [120]. In phase I trial LY2510924 proved to be clinically safe and well tolerated in CRC, pancreatic cancer, and other solid tumors with primary response being 20% of stable disease [121]. A detailed list of modulators of CXCL12–CXCR4/CXCR7 axis in preclinical and clinical studies of gastrointestinal tumors is provided in Table 1. 

Several other molecules are being investigated to target this axis. CXCL12 PEGylated mirror-image l-oligonucleotide (olaptesed-pegol) and CXCR7 inhibitor (CCX733) represent important inhibitors in clinical use for cancer therapies [129]. In vivo, CXCR7 antagonists CCX754 and CXC771 inhibit CRC cell metastasis to the lung [103]. Small molecule inhibitors of CXCR7, such as CCX733 or CCX266, siRNAs, and blocking antibodies have also been examined utilizing in vitro and in vivo experimental models [130]. The CXCR7 antagonists CCX733, CCX754 and CCX771 have been patented by ChemoCentryx as CXCL12–CXCR7 ligands [131,132]. NB1-3, NB2, and NB3 are three nanobodies targeting CXCR7, which can substitute for CXCL12. Amongst these nano-antibodies, binding of CXCL12 to CXCR7 can be partially inhibited by NB1 but not NB2. However, NB3 can inhibit the binding completely by inhibiting CXCL12 recruitment to β-arrestin2 [133]. No pre-clinical or clinical studies using CXCR7 monoclonal antibodies in cancer therapeutics have been reported thus far. Moreover, targeting CXCR4 or CXCR7 alone is not sufficient for inhibiting CXCL12-mediated pro-metastatic responses suggesting that compensatory pathways induced by these receptors can allow tumor escape. Thus, both receptors will likely need to be blocked for effective inhibition of the CXCL12–CXCR4/CXCR7 axis. 

There are rare studies focused on the therapeutic efficacy of CXCR4/CXCR7 inhibitors in gastrointestinal cancers. Although CXCR4 expression is a prognostic factor for several gastrointestinal tumors, CXCL12–CXCR4/CXCR7 axis appears to act as tumor promoter and not tumor initiator. We have earlier demonstrated that ADAM17, the metalloprotease responsible for releasing EGFR ligands, mediates CXCL12–CXCR4 transactivation of EGFR and colonic tumor development [134]. In recent studies, we established that CXCR4 antagonist MSX-122 (substituted pyrimidine-2-amine) blocked CXCL12-induced EGFR transactivation and colon cancer progression in azoxymethane-induced mouse model [126]. We think that MSX-122 might serve as an effective chemo preventive agent in patients with increased risk of colon cancer. MSX-122 is bioavailable and well tolerated in a phase1b clinical trial [126].

Blockade of the CXCL12–CXCR4/CXCR7 axis has provided encouraging results with respect to immunotherapy of glioblastoma [135], ovarian cancer [136], multiple myeloma [137], acute myeloid leukemia [138], cervical cancer [139], as well as relapsed or refractory multiple myeloma [140]. There are no reports, however, of blockade of this axis to synergize with chemotherapy or immunotherapy for gastrointestinal cancers. Most gastrointestinal solid tumors are not suppressed by single agent checkpoint inhibitor therapy. We speculate, however, that colon cancers might be suppressed by combination therapy involving drugs targeting immunosuppressive CXCL12–CXCR4/CXCR7 axis. Future studies should be undertaken to target CXCL12–CXCR4/CXCR7 axis in combination with chemotherapy or immunotherapy to treat advanced gastrointestinal cancers.

In addition, combination of targeted therapies and silencing of genes by small interfering RNA (siRNA) and microRNAs (miR) appears to be an attractive approach to silence or inhibit expression of oncogenes [141]. However, only few clinical trials using siRNA in humans are reported because of problems associated with their delivery [142]. Majority of aberrantly expressed miRs targeting CXCR4 in a wide range of cancers are tumor suppressors, for example, in CRC miR-126 [85,143,144,145], miR-133b [146], miR-193a-5p [147], and miR-622 [148] are tumor suppressors, whereas miR-125b is the only overexpressed onco-miR [149]. MiR-126 and miR-133b hold prognostic value in terms of predicting patient outcome in CRC [144,146], miR-139 in gastric cancer [150], miR-622 in hepatocellular carcinoma [151], and miR-494 in pancreatic cancer [152]. Future therapies in the field of colorectal cancer may also involve genome editing technologies to remove harmful “driver mutations” and insert “preventive mutations” using zinc-finger nucleases, transcription-like effector nucleases, and short palindromic repeat-associated nuclease 9 [153] in CXCL12–CXCR4/CXCR7 axis and its modulators.

## 7. Conclusions

CRC is amongst the leading causes of cancer related deaths worldwide. Chemokine CXCL12 and its receptors CXCR4 and CXCR7 play an important role in regulation of homeostasis under normal physiological conditions. In CRC and other gastrointestinal cancers elevation of this axis lead to progression and metastasis because of altered signaling with unfavorable disease outcome and poor survival of patients. CXCR4 and its antagonists are tried in several clinical trials for the treatment of CRC and other gastrointestinal cancers but with very limited success. So far in CRC, CXCR7 antagonists are only tried in animal models with no clinical data. Antagonists of CXCL12–CXCR4/CXCR7 axis along with chemotherapy or immunotherapy should be tested as combination therapy in translational studies for the treatment of CRC.

## Figures and Tables

**Table 1:** Modulators of CXCL12–CXCR4/CXCR7 axis in preclinical and clinical studies of gastrointestinal cancers.

Inhibitor/Antagonist	Target	Cell-Line/Disease	Biological Effect Monitored/Clinical Trial Number	Trial Phase	References
**Preclinical Studies:**					
CCX754 and CCX771	CXCR7	Murine C26 colon carcinoma	40% Reduction in lung metastases/NS effect in Liver		[103]
		Human HT29 colorectal adenocarcinoma	50% Reduction in lung metastases/NS effect in Liver		[103]
LY2510924	CXCR4	Colorectal carcinoma	Inhibition of tumor growth		[120]
CTCE-9908	CXCR4	Esophageal cancer	Reduction in metastatic spread and primary tumor growth		[122]
TN14003	CXCR4	Pancreatic cancer	Inhibition of migration and invasion		[123]
Plerixafor (AMD3100)	CXCR4	AGS and kato III Gastric Cancer (GC) Cell lines	Inhibition of GC cell invasion		[124]
AMD3100	CXCR4	BGC-823, and SGC-7901 human GC cell lines	Enhances docetaxel cytotoxicity in vitro		[125]
MSX-122	CXCR4	Apc+/Min mice	Blocked EGFR transactivation by CXCL12 and reduced tumor burden		[126]
**Clinical** **Studies:**					
AMD3100 (+Cemiplimab)	CXCR4	Metastatic pancreatic cancer	NCT04177810	Recruiting	ClinicalTrials.gov
AMD3100	CXCR4	Pancreatic cancer	NCT02695966	Completed Phase1	ClinicalTrials.gov
AMD3100	CXCR4	Colorectal, Pancreatic cancer	NCT03277209	Terminated phase1	ClinicalTrials.gov
AMD3100	CXCR4	Colorectal, Pancreatic cancer	NCT02179970	Completed Phase1	ClinicalTrials.gov
MSX-122	CXCR4	Refractory metastatic or Locally advanced solid tumors	NCT00591682	Suspended Phase1	ClinicalTrials.gov
Motixafortide (BL-8040), (+Pembrolizumab), Chemotherapy of Onivyde	CXCR4	Metastatic pancreatic adenocarcinoma	NCT02826486	Active Phase 2a	[127] ClinicalTrials.gov
BL-8040 or BKT140, Multiple immunotherapy-based treatment combinations	CXCR4	Metastatic pancreatic ductal adenocarcinoma	NCT03193190	Recruiting (Phase1b/2)	ClinicalTrials.gov
BL-8040 or BKT140, Multiple immunotherapy-based treatment combinations	CXCR4	Metastatic gastric and esophageal cancer	NCT03281369	Recruiting Phase1b/2	ClinicalTrials.gov
BL-8040 or BKT140 (+Pembrolizumab)	CXCR4	Metastatic pancreatic cancer	NCT02907099	Active Phase2b	ClinicalTrials.gov
BL-8040 (+Cemiplimab)	CXCR4	Pancreatic cancer	NCT04543071	Recruiting Phase 2	ClinicalTrials.gov
Olaptesed pegol (NOX-A12) alone or(+Pembrolizumab)	CXCL12	Colorectal and pancreatic cancer	NCT03168139	Completed Phase1/2	[128] ClinicalTrials.gov
LY2510924	CXCR4	Colorectal and pancreatic cancer	Dose escalation and dose confirmation	Phase1	[121]

## Data Availability

Not Applicable.
